# Supplementary material for: Content-rich biological network constructed by mining PubMed abstracts
Source: BMC Bioinformatics. 2004 Oct 8;5:147. doi: 10.1186/1471-2105-5-147 (PMC528731; doi:10.1186/1471-2105-5-147)
Supplement: Additional File 2 — The original results of the above study (non-essential files are deleted to keep the file size under the limit set by BMC bioinformatics). [file 1471-2105-5-147-S2.bz2 › chilibotAdditionalFile2/dip10/438ID9430724E10383/html/CCR4_CCL22.html]

 


 **CCR4** and **CCL22** 
  
Found 85 abstracts in PubMed, retrieved 10.  
 

 What does Google say? 
 PDF only 
| .edu only 

---

**Interactive relationship** (e.g. stimulation, inhibition, etc)

**Neutral relationship**- Since the ligand of the TARC and MDC  [ **CCL22** ]  is  **CCR4** , which is specifically expressed on Th2 type T cells, the production of these CC chemokines is likely to play important roles in the T cell and B cell interaction.  Ref: 12715916 J Med Dent Sci, 2003
- The helper Th 2 cell attracting chemokines thymus and activation regulated chemokine TARC and macrophage derived chemokine  [ **CCL22** ]  MDC  [ **CCL22** ]  are ligands for the chemokine receptor  **CCR4** .  Ref: 12645858 J Clin Immunol, 2003
- However, T cells which are CD4 CD152  **CCR4**  migrate selectively in vitro in response to the chemokine  **CCL22**  specific for the receptor  **CCR4**  produced by the malignant B cells and are always seen amongst the malignant cells in bone marrow and lymph nodes from B CLL patients.  Ref: 12688308 Leuk Lymphoma, 2003
- Moreover,  **CCR4**  and CXCR6 were preferentially induced in T cells activated by mDC  [ **CCL22** ]  and pDC, respectively.  Ref: 12645946 Eur J Immunol, 2003
- Surprisingly, only the  **CCL22**  analogue with the biotin in position K66 constituted a suitable staining reagent for  **CCR4**  positive cells.  Ref: 12857385 J Biomol Screen, 2003
- CC chemokine receptor 4  [ **CCR4** ]   **CCR4**  has been identified as a specific receptor for both thymus and activation regulated chemokine TARC and macrophage derived chemokine  [ **CCL22** ]  MDC  [ **CCL22** ] , and is preferentially expressed on Th2 cells.  Ref: 12757454 Allergy, 2003
- Three chemokines in particular, CXCL12 SDF 1, CCL17 TARC, and  **CCL22**  MDC  [ **CCL22** ] , recently have been shown to be strong and rapid activators of platelet aggregation and adhesion after their binding to platelet CXCR4 or  **CCR4** , when acting in combination with low levels of primary agonists.  Ref: 12851650 Microcirculation, 2003

**Non-interactive relationship** (e.g. studied together, co-existance, homology, etc.)

- Accumulation of  **CCR4**  expressing CD4 T cells and high concentration of its ligands TARC and MDC  [ **CCL22** ]  in bronchoalveolar lavage fluid of patients with eosinophilic pneumonia.  Ref: 12757454 Allergy, 2003
- We analyzed the functional role of macrophage derived chemokine  [ **CCL22** ]  MDC  [ **CCL22** ] , a potent mononuclear cell chemoattractant, during the progression of anti glomerular basement membrane GBM antibody Ab GN, a model of crescentic GN in the WKY rat, and whether the effects of MDC  [ **CCL22** ]  were dependent on its receptor  **CCR4** .  Ref: 12651599 Am J Pathol, 2003
- BACKGROUND Macrophage derived chemokine  [ **CCL22** ]  MDC  [ **CCL22** ]  is a Th2 type chemokine and its receptor CC chemokine receptor 4  [ **CCR4** ]   **CCR4**  is preferentially expressed on Th2 cells.  Ref: 12670721 J Dermatol Sci, 2003
- CONCLUSIONS Our data suggest that Th2 cells, which express  **CCR4**  and its ligands TARC and MDC  [ **CCL22** ] , contribute to the pathogenesis of EP in the lung.  Ref: 12757454 Allergy, 2003
- There was a significant correlation between the number of  **CCR4**  CD4 T cells and the levels of TARC, MDC  [ **CCL22** ] , and IL 5 in BALF of patients with EP.  Ref: 12757454 Allergy, 2003
- In this study, we also found that MDC  [ **CCL22** ]  activity on Mo Mphi in this GN was at least partly dependent on a new variant of  **CCR4** .  Ref: 12651599 Am J Pathol, 2003
